# Impact of Particle Size on Performance of Selective Laser Sintering Walnut Shell/Co-PES Powder

**DOI:** 10.3390/ma14020448

**Published:** 2021-01-18

**Authors:** Yueqiang Yu, Minzheng Jiang, Suling Wang, Yanling Guo, Ting Jiang, Weiliang Zeng, Yu Zhuang

**Affiliations:** 1 College of Mechanical Science and Engineering, Northeast Petroleum University, Daqing 163318, China; yqyu@nepu.edu.cn (Y.Y.); wangsuling@nepu.edu.cn (S.W.); tjiang@nepu.edu.cn (T.J.); 2 College of Mechanical and Electrical Engineering, Northeast Forestry University, Harbin 150040, China; yuyueqiang@nefu.edu.cn; 3 Research and Development Center of 3D Printing Material and Technology, Northeast Forestry University, Harbin 150040, China; 4 School of Mathematical Sciences, Harbin Normal University, Harbin 150080, China; zengwl@hrbnu.edu.cn

**Keywords:** additive manufacturing, selective laser sintering, walnut shell, particle size, sintering quality

## Abstract

The agricultural and forestry waste walnut shell and copolyester hot-melt adhesives (Co-PES) powder were selected as feedstock. A kind of low-cost, low-power consumption, and environmentally friendly walnut shell/Co-PES powder composites (WSPC) was used for selective laser sintering (SLS). Though analyzing the size and morphology of walnut shell particle (≤550 μm) as well as performing an analysis of surface roughness, density, and mechanical test of WSPC parts with different particle sizes, results showed that the optimal mechanical performance (tensile strength of 2.011 MPa, bending strength of 3.5 MPa, impact strength of 0.718 KJ/m^2^) as walnut shell powder particle size was 80 to 120 μm. When walnut shell powder particle diameter was 120 to 180 μm, the minimum value of surface roughness of WSPC parts was 15.711 μm and density was approximately the maximum (0.926 g/cm^3^).

## 1. Introduction

Additive manufacturing is a processing and manufacturing method, based on the digital model file, the fusible materials are accumulated and solidified layer by layer, so as to produce solid parts [1]. Compared with traditional methods of subtractive manufacturing, additive manufacturing is a manufacturing method of “bottom-up” materials accumulation [2]. This method has a high degree of freedom in product design and manufacturing, thus easily fabricating customized production. Therefore, it is mainly used for iterative design, rapid prototype manufacturing and verification, and producing industrial parts [3,4,5]. Selective laser sintering (SLS) [6], an additive manufacturing technology, was proposed by C.R. Decker [7]. SLS has some strengths over other additive manufacturing techniques, for instance, materials can be reused and high-precision parts can be fabricated [8]. At present, the powder materials applied in SLS technology mainly focus on metals composites [9,10,11], ceramics composites [12,13], and polymers composites [14,15,16,17]. Because most SLS materials have high cost, the research and development of new powder materials suitable for SLS technology have become a hot spot in current research.

Biomass composite materials mainly consist of the remains of agriculture and forestry, such as wood, straw, bamboo, rice husk and fruit shell, polymers, and micro additives [18]. Considering the advantages of biomass composites, such as simple preparation process, low price, good molding performance, and texture [19,20], researchers used an environmental, recycled, and low-cost biomass composite as a new material of SLS technology [21]. Zhang et al. took pine powder of 45–96 μm as raw material and studied parameters such as different material ratios and laser energy. When the mass ratio of pine powder to Co-PES was 1:4, the sintered parts had good toughness, and when the energy density was 0.312 J/mm^3^, the sintered parts had good forming effect [22]. Zhang et al. used pine powder of 45–96 μm as raw material to conduct SLS experiment on wood powder/PES powder with different contents of carbon nanotubes. When the content of carbon nanotubes was 0.1%, the mechanical properties of wood powder/PES parts were highest [23]. Zeng et al. took birch powder as raw material to prepare laser-sintered birch/Co-PES parts and conducted wax-infiltrated treatment. The results showed that the mechanical strength and surface quality of the treated birch/Co-PES parts were significantly improved, while the porosity was significantly reduced [24,25]. Zhao et al. used bamboo powder of 96 μm as raw material to prepare laser-sintered bamboo powder/Co-PES parts with different material ratios, and the mechanical strength of the resin-infiltrated bamboo powder/Co-PES parts was greatly improved [26]. Then, they took bamboo powder of 120 μm as raw material to prepare the laser-sintered bamboo powder/copolyamide parts, and the tensile strength was significantly improved compared with bamboo powder/Co-PES parts [27]. Zeng et al. used rice husk powder of 300 μm as raw material to prepare laser sintered rice husk/Co-PES parts, and carried on the wax-infiltrated post-processing, the bending strength of wax-infiltrated parts are significantly increased [28]. Yu et al. took walnut shell powder of 58–96 μm as raw material to prepare WSPC powder, and verified the feasibility of using WSPC powder for SLS by single-layer sintering experiment [29]. Through the study of different material ratio, the mechanical properties of WSPC parts were improved [30], and it was determined that when the volume fraction of walnut shell powder reached 40%, the warping deformation of WSPC parts was minimum and the dimensional accuracy was highest [31]. WSPC powder with 40% walnut shell was used to conduct SLS test by using five-factor and four-level orthogonal experimental design method, and the optimal process parameters were determined by using comprehensive weighted scoring method [32]. The optimal WSPC parts were treated with wax infiltration, and the post-treatment process was optimized. The optimized WSPC wax-infiltrated parts were conducted with investment casting to obtain metal parts with stable structure and smooth surface [33]. The previous research on SLS biomass composites mainly focused on the molding mechanism of SLS, the composition of biomass composite powder materials, laser sintering process, and post-treatment process. In the SLS process, it was found that the particle size, geometric shape and distribution of biomass powder particles had significant effects on the SLS process of biomass composite powder and the properties of sintered parts. However, the study on the effect of biomass powder particle size on the properties of sintered parts had not been reported.

Walnut shell has a hard and dense texture [34]. Compared to bamboo, wood, and rice husk, it has unique advantages, such as ease of crushing and different particle diameters, such that it easily meets the requirements of powder particle sizes of materials by SLS. Hence, this paper selected walnut shell powder as raw material of SLS. The ingredients of different particle-sized walnut shell powder and Co-PES powder were systematically studied, additionally, the sintering mechanism of walnut shell composites (WSPC) of different particle-sized walnut shell powder were deeply analyzed, and then the density, surface quality and mechanical performance were evaluated. It lays a foundation for regulating the performances of WSPC parts.

## 2. Experimental

### 2.1. Composites Preparation

**Figure 1:**
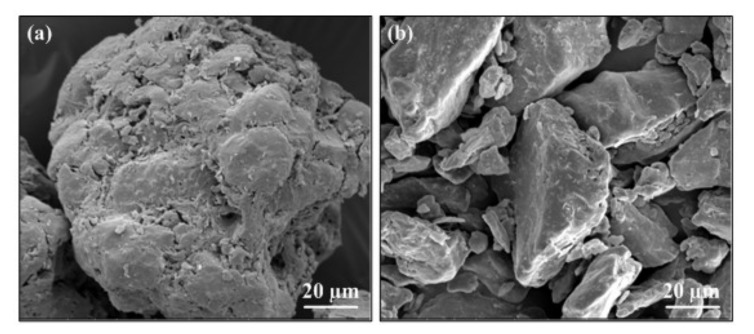
Morphologies of powder particles: (**a**) walnut shell powder particles (**b**) Co-PES powder particles.

WSPC composites were composed of walnut shell particles, Co-PES particles as well as micro-additive. Walnut shell particles (Ding Sheng Corundum Abrasives Ltd., Zhengzhou, China) were yellow-brown porous-surfaced powder, particle diameter (≤550 μm) in Figure 1a. Co-PES particles (Tiannian Material Technology Ltd., Shanghai, China) mainly consist of 1,4 butanediol, m-phthalic acid and dimethyl terephthalate. (white powder, apparent density 0.7 g·cm^−3^; particle diameter ≤58 μm, melt flow rate 35 g/10 min at 160 °C, viscosity 350 Pa·s at 160 °C) in Figure 1b. Microauxiliaries were mainly light stabilizers and lubricants. The light stabilizers (density 1.18 g/cm^3^, melting point 80 °C) was purchased from Zhenhai Jianghua Chemical Industry Ltd. (Ningbo, China), and the lubricant (zinc stearate, density 1.095 g/cm^3^, melting point 125 °C) was obtained from Tianjin Guangfu Fine Research Institution (Tianjin, China).

Waste walnut shell was broken into walnut shell powder particles of different sizes by crusher. Crush the walnut shell powder particles by a winnower for air separation to remove the impurity and diaphragm of the walnut shell powder particles, and polish them by cylinder polishing machine to get rid of the convex peak on the surface of powder particles. After steam washing for 1–3 h by steam washing machine, and drying at a temperature of 85 °C to 90 °C for 20 h in the dryer, finally filtering through vibrating screen, different particle size range of walnut shell powder was obtained as shown in Table 1. After crushing, polishing, steaming, washing and filtering, the obtained walnut shell powder was dehydrated at a temperature of 105 °C for 3.5 h in incubator and weighed every one hour to keep the mass unchanged. Then, the dried walnut shell powder was mixed with Co-PES powder in a 1:1.5 ratio [31] by volume in different ratios (Table 1), using an SHR50A high-speed mixer from Hongji Machinery Ltd. (Zhangjiagang, China). Microauxiliaries whose volume fraction was 2% was added in order to prefer powder dispersity. Microauxiliaries include light stabilizers and lubricant. Light stabilizers enable WSPC powders to exclude or slow down photochemical reactions under laser radiation, prevent or delay the photoaging process, and extend the life of WSPC products. The lubricant can reduce the friction coefficient between the mixed powder, so that the mixed powder has a good dispersion, and at the same time, it can reduce the intermolecular force of the blend and weaken the interface effect, playing a certain plasticizing effect. The powder was blended not higher than 30 °C for 15 min at low velocity, and afterwards for 5 min at high velocity to make the powder uniform. Finally, it was cooled to room temperature.

### 2.2. Forming Principle and Method of SLS

**Figure 2:**
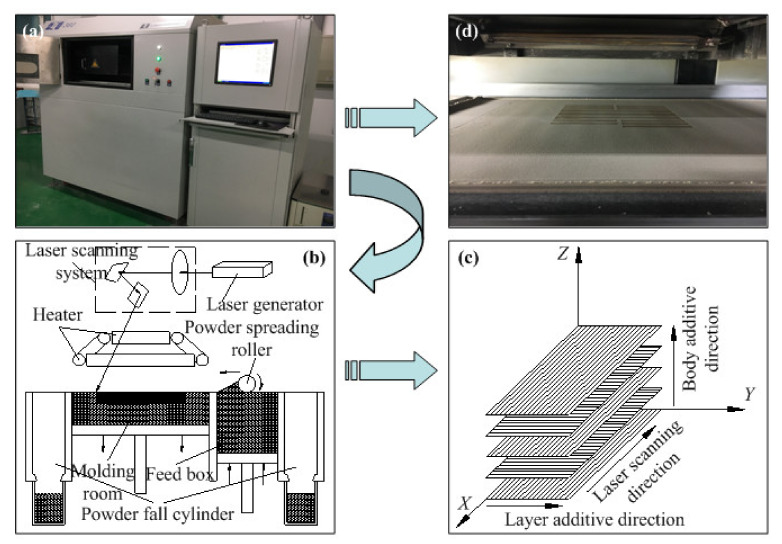
Process of WSPC for SLS: (**a**) AFS-360 rapid prototyping equipment (**b**) schematic diagram of SLS (**c**) sintering method (**d**) sintering state.

The rapid prototyping equipment (AFS-360, Beijing Longyuan Technology Ltd., Beijing, China) used in the experiment mainly composed of powder spreading system, laser scanning system, working cylinder (Figure 2a), heat control system, and CO_2_ laser generator (laser power of 55 W and wavelength of 10.6 μm). Based on the CAD model, through depositing repeatedly a thin layer of fusible powder with laser beam in a repetitive manner, and then a three-dimensional solid object is obtained. The schematic diagram of process is displayed in Figure 2b. Set adjacent samples distance as 5mm, aiming to stop thermal interference. Figure 2c,d shows sintering method and experiment. Table 2 shows the processing parameters for SLS.

### 2.3. Characterization and Test

Powder particle size analysis: a certain amount of walnut shell powder particles were weighed, then the obtained particles were dehydrated for 3.5 h at 105 °C in an incubator (Beijing Longyuan Technology Ltd., Beijing, China) to remove their moisture. Based on the dry measurement principle, powder particles of different sizes and good dispersion were tested by S3500 laser particle size analyzer (Microtrac Inc., Montgomeryville, PA, USA). The test results were repeated for 3 times, and the average value was obtained. The particle sizes and particle size distribution curves of walnut shell powder were plotted.

Walnut shell powder, Co-PES powder, surfaces and cross-sections of WSPC parts were first sputtered with gold because the specimens were non-conductive, and then they were observed using a scanning electronic microscopy (SEM) (FEI Quanta200, Dutch company, Rotterdam, The Netherlands).

Density test: the density of walnut shell powder and WSPC powder was tested by ST-2106A apparent density tester (Xiamen Ester Instrument Co., Xiamen, China). The test was identical with the ISO60:1977 standard [35], repeated for 3 times. The average value of the test results was taken to obtain the density of walnut shell powder of different particle sizes and WSPC powder.

Using an electronic balance and vernier caliper, the mass and dimensions of the WSPC parts were measured. The test was repeated three times and the test results were averaged. The density was calculated using the relation [36]:

(1) $$\rho =\frac{W}{l\times b\times h}$$

where *W* denotes the mass of parts (g), *l* represents the length of parts (mm), *b* is the width of parts (mm), and *h* denotes the thickness of parts (mm).

Cube specimens were selected as roughness test samples, the size of 20 mm × 20 mm × 20 mm, using VK-X1000 shape measurement laser microscopic system (Keyence Corporation, Osaka, Japan) to test the surface roughness of WSPC parts with different walnut shell powder particle sizes. The test was repeated three times. Their three-dimensional structure figures and the surface roughness values Ra and Rz were obtained.

Through a tensile testing machine (CMT5504, MTS System Ltd., Sunnyvale, CA, USA) and a TCJ-4 impact testing machine, the mechanical performance of the WSPC parts with different walnut shell powder particle sizes was measured. The test was repeated three times and the test results were averaged value. The tensile strength is identical with ISO527-2 [37]. The gauge length was 50 mm, and the crosshead speed was 5 mm/min. The tree-point bending strength is identical with ISO178 [38]. The span length was 64 mm, and the crosshead speed was 5 mm/min. The U-notched impact strength is identical with ISO179-2 [39]. The span length was 60 mm, and the pendulum impact power was 4 J.

## 3. Results and Discussion

### 3.1. Particle Size Analysis

The particle size of organic filler in SLS process play a strong part in the effect of spread powder. The effect of spread powder directly affects the forming quality of parts. As the walnut shell powder particles are made by mechanical crushing, their shapes are not uniform. Therefore, it is necessary to analyze the influence of the size distribution and shape of walnut shell powder particles on the effect of spread powder.

**Figure 3:**
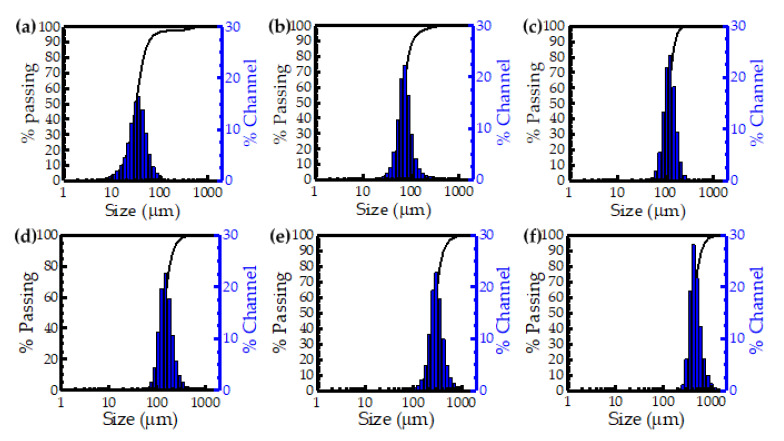
Particle size distribution of walnut shell powder: (**a**) type I (**b**) type II (**c**) type III (**d**) type V (**e**) type IV (**f**) type VI.

**Figure 4:**
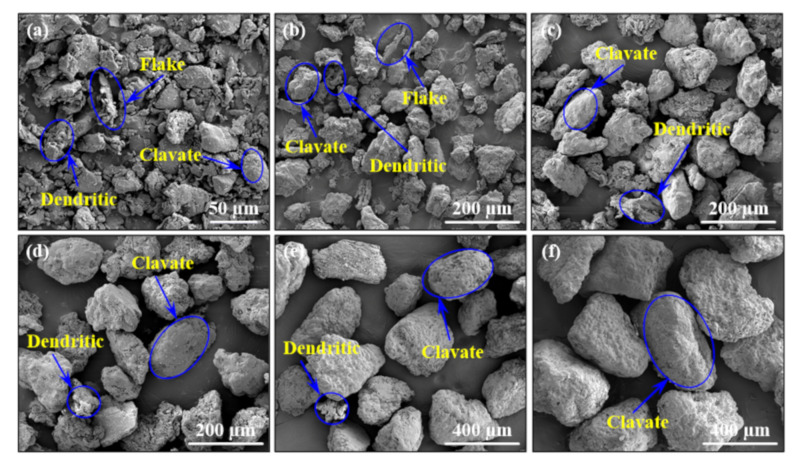
Microscopic morphology of walnut shell powder particle: (**a**) type I, (**b**) type II, (**c**) type III, (**d**) type IV, (**e**) type V and (**f**) type VI.

Figure 3 and Figure 4 show the particle size distribution and microscopic morphology of different walnut shell powder particle sizes. In the Figure 3a,b and Figure 4a,b, type I presented most walnut shell powder particle sizes gathered in 45 μm, and type II showed sizes gathered in 75 μm. These kinds of powder particle shapes were very diverse, like dendritic, flake, and clavate. As shown in Figure 3c–e and Figure 4c–e, type III presented most walnut shell powder particle sizes gathered in 114 μm, type IV showed sizes gathered in 148 μm and type V showed sizes gathered in 290 μm. These kinds of powder particle shapes were relatively simple, only like dendritic and clavate. In Figure 3f and Figure 4f, type VI presents most walnut shell powder particle sizes gathered in 439 μm, powder particle shape is the simplest, only like clavate. Therefore, it can be concluded that the smaller the walnut shell powder particles sizes were, the more the particle shape types were, and the more complex the particle system was. However, with increasing the walnut shell powder particles sizes, the particle shape types decreased, and the particle system became simple. In the process of SLS, flake and dendritic powder particles were inconvenient for spreading powder, but clavate-shaped particles closer to spherical particles, which was advantageous to spread powder. Importantly, the walnut shell powder particles should not be too big, and in type VI, large powder particle size led to big dimension deviation of sintered parts under process parameters in Table 2. Hence, type VI was ignored in the follow-up study.

### 3.2. Surface Quality

Particle size of organic filler and dispersion in amorphous polymer matrix play an important role in the surface quality of WSPC parts in SLS process. The surface quality of parts directly affects the usability of parts. Therefore, the surface morphologies of SLS parts need to be observed to study the influence of walnut shell powder of different particle sizes on the surface morphologies of WSPC parts.

**Figure 5:**
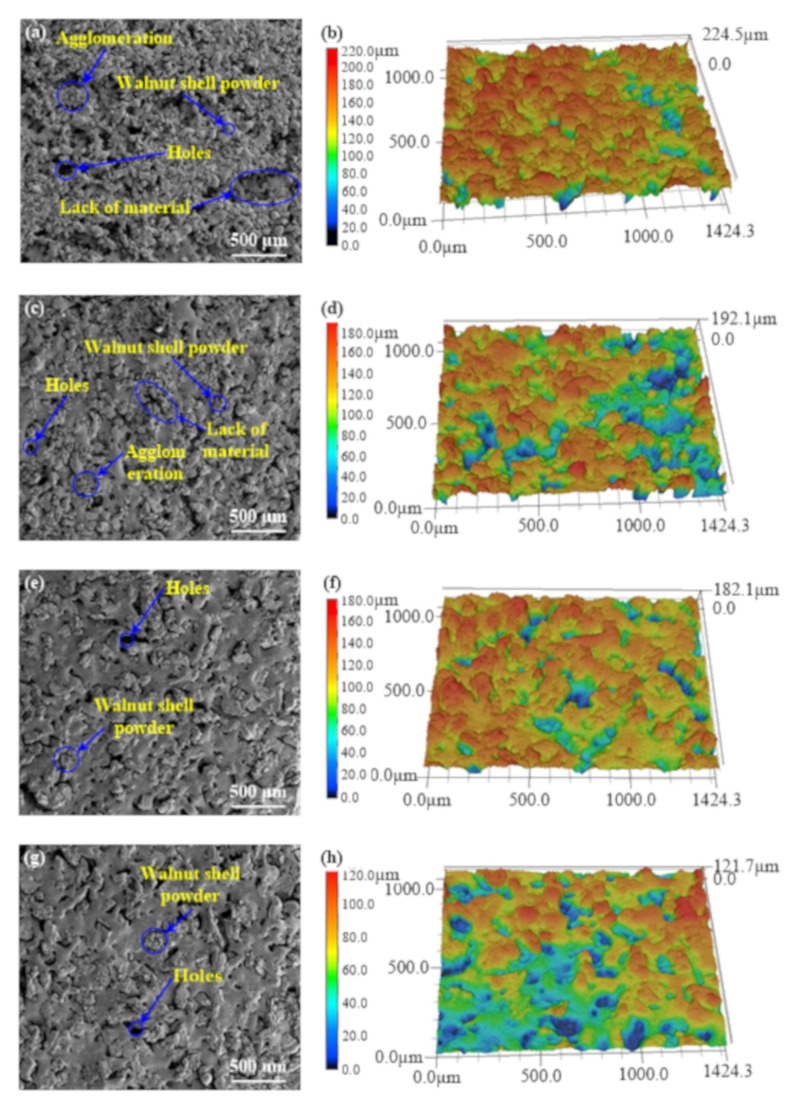

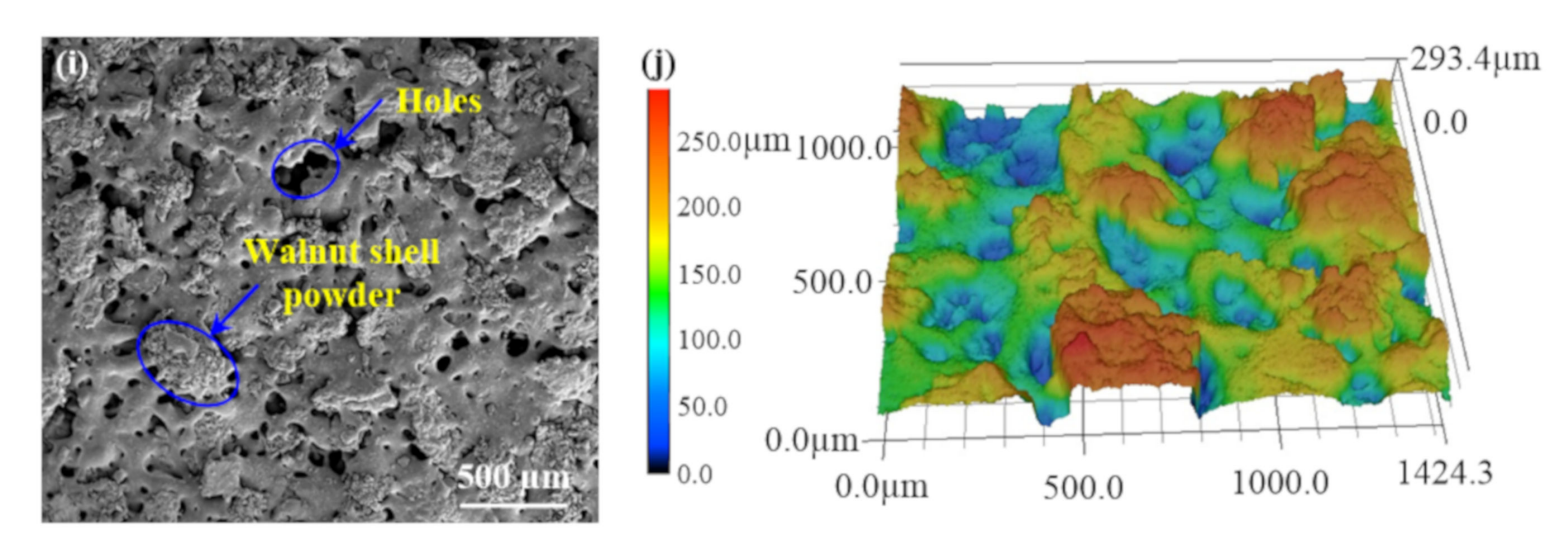
Micro-morphologies and three-dimensional morphologies of WSPC parts with different walnut shell powder particle sizes. (**a**) and (**b**) type I, (**c**) and (**d**) type II, (**e**) and (**f**) type III, (**g**) and (**h**) type IV, (**i**) and (**j**) typeV.

The upper surface of the sintered sample shown in Figure 2c is used as the test surface to obtain the microstructure and three-dimensional morphology of the upper surface of WSPC parts with walnut shell powder of different particle sizes in Figure 5. The roughness values of the upper surface are shown in Table 3. Figure 5 shows micro-morphologies and three-dimensional morphologies of WSPC parts surface with different walnut shell powder particle size. Table 3 presented surface roughness of WSPC parts. In Figure 5a,b, type I present there were many holes, powder particles agglomeration and amount of material loss zones on the surface of WSPC parts, thus the surface was very rough. Surface roughness Ra and Rz were 27.567 μm and 177.338 μm. The main reason is in typeIthe walnut shell powder particle size is small, surface area is large, the adhesion force between particles is large, causing the powder particles adhesion on the powder spreading roller, widespread lack of material, easy to make the powder particles agglomeration to form larger particles. Fused Co-PES powder particles cannot fill pores between larger particles, forming holes on surface of the WSPC sintering parts. Larger particles caused by agglomeration, loss of material area and the holes result in a big distance between baseline and points on the measured surface profile, forming the peaks and troughs, even a large number of peaks and troughs, therefore, in type I WSPC parts show fairly rough upper surface and the surface quality is poor. In Figure 5c,d, type II showw that there were many holes and powder particle agglomeration on the surface of WSPC parts, but the material loss zone was small. Its surface was still rough because of material loss, surface roughness Ra and Rz were 23.973 μm and 171.641 μm. The main reason is in typeIIthe walnut shell powder particle size increases, the surface area decreases, and adhesion force between particles decrease, the powder particles adhesion on the powder spreading roller decrease, forming a small area of the material lack, agglomeration of powder particles decreases, but fused Co-PES powder particles cannot fully fill pores between larger particles, forming holes on surface of the WSPC parts. A few particles, because of agglomeration, loss of material area, and the holes, cause a big distance between baseline and points on the measured surface profile, forming the peaks and troughs, but a small number of peaks and troughs. Therefore, in type II WSPC parts show rough upper surface and the surface quality is poor. From Figure 5e–h, types III and IV present that there were few holes and no material loss zone on the surface of WSPC parts. Compared with type IV, type III presented more evenly particles distribution. Because there was no lack of material zone, walnut shell powder particles did not stand out obviously. Type IV presented a flat surface, and its surface roughness was minimum. Ra and Rz were 15.711 μm and 99.721 μm. In type III, walnut shell powder particles stood out obviously, the surface was relatively rough, Ra and Rz were 21.271 μm and 161.617 μm, respectively. The main reason is in type III and type IV walnut shell powder particle size increases, the particle surface area decreases, and adhesion force between particles continue to decrease [40], cannot cause powder particles adhesion on spread powder roller and lack of material. At the same time, nearly no agglomeration phenomenon of the powder particles was observed, and fused Co-PES powder particles almost fully fill pores between larger particles, forming a few holes on surface of the WSPC parts. A few holes and outstanding walnut shell powder particles cause a big distance between baseline and points on the measured surface profile, forming the peaks and troughs. Therefore, in type III WSPC parts show rough upper surface and the surface quality is poor. In type IV only a few holes also cause a big distance between baseline and points on the measured surface profile, forming the peaks and troughs, but a small number of peaks and troughs, therefore, type IV WSPC parts show smooth upper surface and the surface quality is good. From Figure 5i,j, type V show there was no lack of material but more holes on the surface area, and the walnut shell powder particles was distributed unevenly and stood out obviously. Type V presented very rough surface, and its surface roughness was maximum. Ra and Rz were 36.028 μm and 204.952 μm. When the particle size of walnut shell powder increased, the surface area of powder particles decreased and the adhesion force among particles decreased, so the agglomeration and adhesion of powder particles did not occur on the powder spreading roller, causing no material loss zone on the surface of parts. However, the size difference between walnut shell powder particles and Co-PES powder particles is too large, thus, walnut shell powder particles cannot be distributed evenly on the powder bed, which leads to its uneven distribution and standing out obviously on the surface. Large size of walnut shell powder particles make the molten Co-PES powder particles not completely fill the pores between powder particles, causing large holes on the surface of WSPC parts.

### 3.3. Morphologies

Particle sizes of organic filler and dispersion in amorphous polymer matrix have a significant influence on the inner structure of WSPC parts in SLS process. The degree of compactness of the internal structure of parts directly affects their performance. Therefore, the inner morphologies of SLS parts need to be observed to explore the effect of walnut shell powder of different particle sizes on the morphologies of WSPC parts.

**Figure 6:**
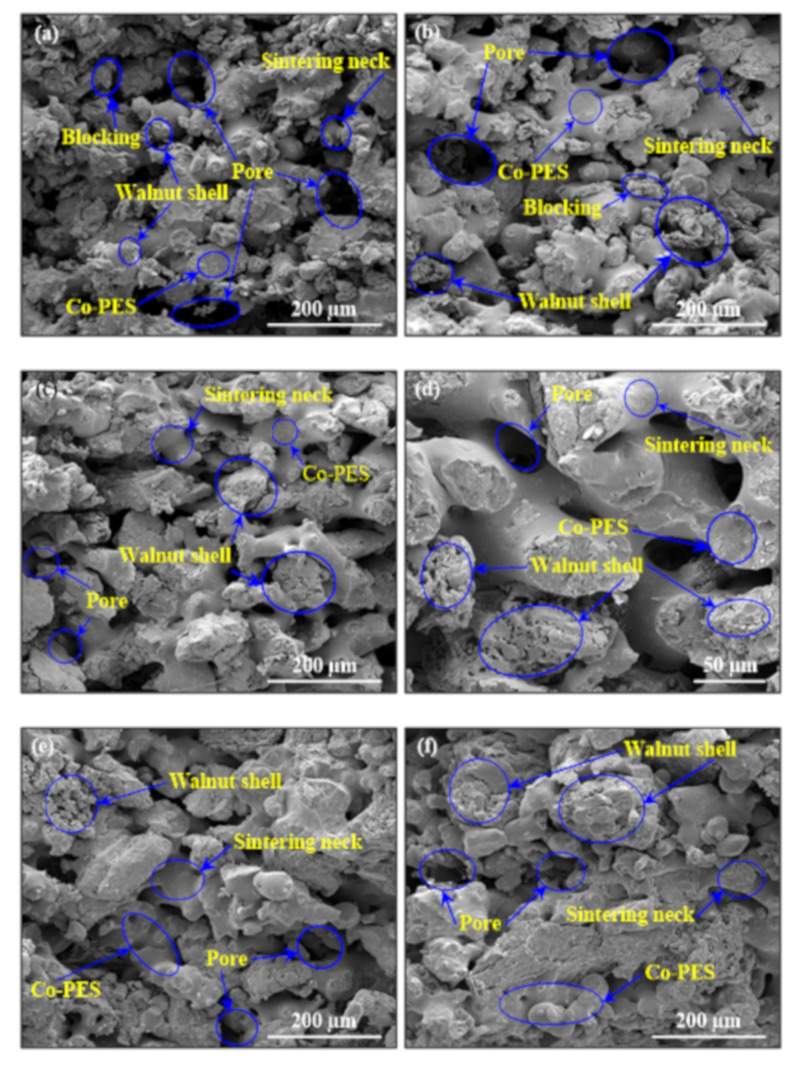
SEM figures of sections of WSPC parts with different walnut shell powder particle sizes: (**a**) type I, (**b**) type II, (**c**) and (**d**) type III, (**e**) type IV and (**f**) type V.

Figure 6 shows the SEM figures of sections of WSPC parts with different particle-sized walnut shell powder. From Figure 6a,b, it can be seen that types I and II presented the majority of walnut shell powder particles exposed and could not be fully wrapped by Co-PES matrix, causing low interface bonding strength and forming a few small sintering necks, but sintering necks were blocked by the self-contact of walnut shell powder particles. The size and quantity of the inner pores of the WSPC parts were more, the density was relatively low. The main reason was walnut shell powder particle size was small and surface energy was big, causing the self-contact. At the same time, the molten Co-PES accelerated the walnut shell powder particles movement, and made them float on the Co-PES matrix surface, leading to the walnut shell powder particles unwrapped. Therefore, the inner pores of the WSPC parts was large, and the density was relatively low.

From Figure 6c,d type III shows that walnut shell powder particles were distributed evenly in the Co-PES matrix, and almost all the walnut shell powder particles were fully wrapped by Co-PES matrix, and the interface bonding strength was strong, forming a lot of sintering necks and a net structure. However, the internal pores of WSPC parts were more, and the density was relatively low. The main reason was walnut shell powder particles blocked the flow of molten Co-PES. The molten Co-PES rearranged and wrapped walnut shell powder particles. The walnut shell powder particles and the molten Co-PES were combined with each other, forming a net structure and locking each other, but there were many internal pores. Therefore, the density was relatively low.

In Figure 6e,f, types IV and V show the walnut shell powder particles could not be fully wrapped by Co-PES matrix. The interface bonding strength was high, but only a few bigger sintering necks formed, causing that walnut shell powder particles in the Co-PES matrix distribution was not uniform, and it was easy to generate a large number of local accumulations in the Co-PES matrix. Type IV presented fewer inner pores and relative higher density. The main reason showed that the size difference between walnut shell powder particles and Co-PES powder particles was large, the molten Co-PES easily flowed and filled the big gaps between walnut shell powder particles, but causing local accumulation of Co-PES matrix. Type V showed bigger inner pores and relative lower density. The main reason showed that the size difference between walnut shell powder particles and Co-PES powder particles was very large, it was hard for the walnut shell powder particles to be uniformly distributed in the powder bed when spreading powder. Meanwhile, the molten Co-PES could not fully wrap walnut shell powder particles and completely fill the big gaps between walnut shell powder particles, and thus it was easy to cause local accumulation of molten Co-PES. Therefore, the inner pores and quantity of the WSPC parts was large, and the density was relatively low.

### 3.4. Density of Parts

Particle sizes of organic filler and dispersion in amorphous polymer matrix are important factors affecting the density of WSPC parts in SLS process. The density of parts indirectly reflects the degree of compactness of the internal structure of parts. Therefore, it is necessary to test the density of SLS parts to explore the effect of walnut shell powder of different particle sizes on the density of WSPC parts.

**Figure 7:**
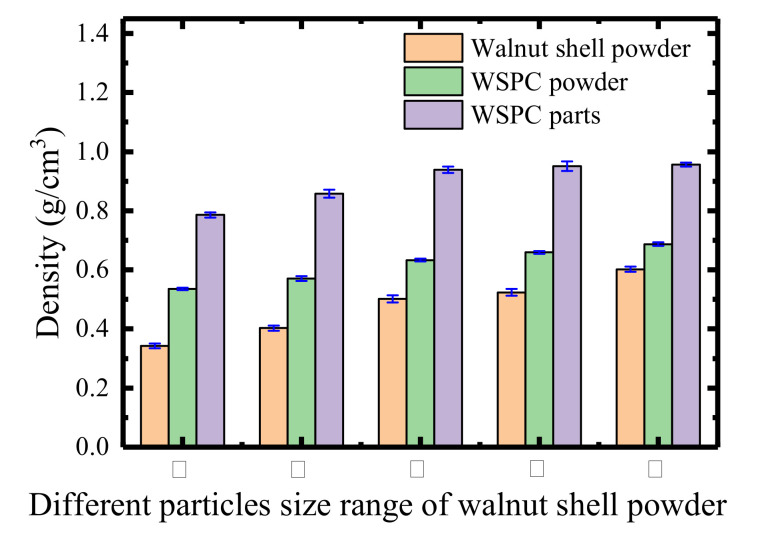
Histograms of density of powder particles and parts.

Figure 7 shows histograms of density of walnut shell powder, WSPC powder and WSPC parts with different walnut shell powder particle sizes. It can be seen from Figure 7, with walnut shell powder particle size increased, the bulk densities of walnut shell powder particle and WSPC powder increased. The main reason is that walnut shell powder particle size increases gradually but Co-PES powder particle size is unchanged, causing the size difference between walnut shell powder particles and Co-PES powder particles gradually increased, and Co-PES powder particles fill the gaps between walnut shell powder particles. Therefore, with walnut shell powder particle size increased, the bulk density of WSPC powder increased. With the increase of particle size of walnut shell powder, the density of WSPC parts first increases and then decreases. The main reason is types I and II show the walnut shell powder particle size is small and molten Co-PES makes walnut shell powder particles move, making walnut shell powder particles float on the surface of the liquid and not be wrapped by molten Co-PES, thus entities cannot be formed. The size difference between walnut shell powder particle and Co-PES powder particle is not big, causing a big gap among the composite powder particles and decreasing bulk density of powder bed, so the internal pores of WSPC parts are more and the compactness is relatively low. Therefore, the density of WSPC parts is low. In type III walnut shell powder particle size is bigger than that of types I and II, with the increase of walnut shell powder particle size, size difference between walnut shell powder particles and Co-PES powder particle is big, thus improving the bulk density of powder bed, and Co-PES powder particle size is small, under the action of laser, sintering rate is fast and melting is sufficient, but walnut shell powder particles hinders the flow of molten Co-PES, not making molten Co-PES completely fill the gap between the walnut shell powder particles, which causes many internal pores of WSPC parts, with a relatively low density. In type IV walnut shell powder particle size is bigger than that of type III, the molten Co-PES easily flows into the gap between walnut shell powder particles, the internal pores of WSPC parts are less. The density is high and reaches maximum, where its value is 0.926 g/cm^3^. In type V the size difference between walnut shell powder particles and Co-PES powder particle is bigger. The bulk density of powder bed is improved, and molten Co-PES easily flows to the gap between the walnut shell powder particles, but molten Co-PES cannot fully wrap walnut shell powder particles and fill a larger gap between the walnut shell powder particles. So, the internal pores of WSPC parts are more and the compactness is relatively low. Therefore, the density of WSPC parts is low.

### 3.5. Mechanical Properties

The interface bonding strength between particle size of organic filler and amorphous polymer matrix plays a strong part in the mechanical properties of parts. The mechanical properties of parts directly affect the usability of parts. Therefore, it is necessary to test the mechanical properties of SLS parts to study the effect of walnut shell powder of different particle sizes on the mechanical properties of WSPC parts.

**Figure 8:**
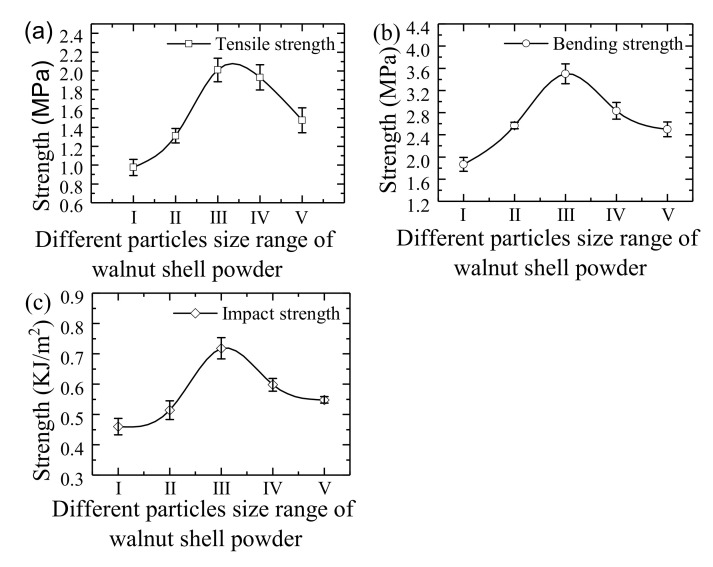
The mechanical properties change curves of WSPC parts with different walnut shell powder particle sizes: (**a**) tensile strength, (**b**) bending strength and (**c**) impact strength.

Figure 8 shows the change curves of tensile strength, bending strength and impact strength of WSPC parts with different walnut shell powder particle sizes. The tensile, bending and impact strengths of WSPC parts show the same change trend, increasing first then decreasing with the increase of walnut shell powder particle size. Type III shows the tensile, bending and impact strength of WSPC parts reach highest, its values are 2.011 MPa, 3.5 MPa and 0.718 KJ/m^2^. The main reason is in type I and type II internal pores and quantity of WSPC parts are large, most of walnut shell powder particles are exposed and cannot be fully wrapped by Co-PES matrix, causing low interface bonding strength, so the tensile, bending and impact strengths are low. Type III shows many inner pores of WSPC parts, but the walnut shell powder particles distributed evenly in the Co-PES matrix, almost all of the walnut shell powder particles are wrapped by Co-PES matrix, forming a net structure and mutual lock. The interface bonding strength between walnut shell powder particles and Co-PES matrix is strong, so the tensile, bending, and impact strengths are high and reach the largest. In type IV internal pores of WSPC parts are small, but walnut shell powder particles in the Co-PES matrix was unevenly distributed, which is easy to generate a large number of local accumulations in the Co-PES matrix, causing stress concentration, so the tensile, bending and impact strengths are low. In type V internal pores and quantity of WSPC parts are larger, the walnut shell powder particles cannot be fully wrapped by Co-PES matrix. The interface bonding strength was stronger, but the walnut shell powder particles in the Co-PES matrix distribution is uneven, which is easy to generate a large number of local accumulations in the Co-PES matrix, causing stress concentration, so the tensile, bending, and impact strengths are low.

## 4. Conclusions

1. Through the analysis of the sizes and shapes of walnut shell powder particles, due to the large particle size and a few types of particle shapes, the melt-viscosity is low, and the spread powder effect is good.
2. After analyzing surface morphologies and surface roughness of WSPC parts, smaller walnut shell powder particles result in more holes on the surface of WSPC parts. Walnut shell powder particles agglomeration and loss of material areas lead to rough surface. Large particles result in large holes on the surface of WSPC parts. At the same time, walnut shell powder particles are distributed unevenly and stand out obviously, which also causes rough surface. When the particle size of walnut shell powder is 120 to 180 μm, there are a few holes on the surface of WSPC parts, and walnut shell powder particles are evenly distributed. Moreover, there is no loss of material area, showing a flat surface, and its surface roughness was minimum. Ra and Rz are 15.711 μm and 99.721 μm.
3. By morphologies analysis, it can be indicated that smaller walnut shell powder particles result in larger and more internal pores of WSPC parts, lower density, smaller sintering necks and lower interface bonding strength. Large walnut shell powder particles tend to cause uneven distribution of walnut shell powder particles in the Co-PES matrix, resulting in a large number of local aggregation phenomenon in the Co-PES matrix, which leads to larger and more internal pores of WSPC parts, and lower density. When the particle size of walnut shell powder is 80–120 μm, the interfacing strength between Co-PES matrix and walnut shell powder particles is the best in WSPC parts. However, when the walnut shell powder particle size is 120–180 μm, the internal pores of WSPC parts are few and density is relatively high.
4. Through density analysis, the density of WSPC parts first increases and then decreases as the walnut shell powder particle size increases. When the walnut shell powder particle size is 80–120 μm, the density of WSPC parts closes to the maximum, and its value is 0.926 g/cm^3^.
5. Mechanical test analysis of WSPC parts shows that the tensile strength, bending strength and impact strength of WSPC parts first increases and then decreases because of increasing walnut shell powder particle size. When the walnut shell powder particle size is 80–120 μm, the mechanical properties of WSPC parts are the best, namely the tensile, bending, and impact strengths reach 2.011 MPa, 3.5 MPa, and 0.718 KJ/m^2^, respectively.

## Figures and Tables

**Table 1:** Particle size configuration of WSPC powder.

Test Number	Walnut Shell Powder Particles (µm)	Co-PES Powder Particles (µm)
I	≤58	≤58
II	58–80	≤58
III	80–120	≤58
IV	120–180	≤58
V	180–380	≤58
VI	380–550	≤58

**Table 2:** The SLS process parameters of WSPC [32].

Laser Power (W)	Scan Speed (mm/s)	Layer Thickness (mm)	Scan Spacing (mm)	Preheating Temperature (°C)	Processing Temperature (°C)
12	2000	0.15	0.2	80	75

**Table 3:** Surface roughness of WSPC parts.

Test Number	Surface Roughness Ra (µm)	Surface Roughness Rz (µm)
I	27.567	177.338
II	23.973	171.641
III	21.271	161.617
IV	15.711	99.271
V	36.028	204.952

## Data Availability

The data presented in this study are available on request from the corresponding authors.
